# A comparative study of irrigation versus no irrigation during burr hole craniostomy to treat chronic subdural hematoma

**DOI:** 10.1186/s12893-017-0295-x

**Published:** 2017-09-11

**Authors:** Qiang-Ping Wang, Ye Yuan, Jun-Wen Guan, Xiao-Bing Jiang

**Affiliations:** 1 Department of Neurosurgery, Union Hospital, Tongji Medical College, Huazhong University of Science and Technology, 1277 JieFang Avenue, Wuhan, 430022 China; 2 Department of Neurosurgery, West China Hospital, Sichuan University, Chengdu, 610041 China

**Keywords:** Chronic subdural hematoma, Burr hole, Drainage, Irrigation, Outcome

## Abstract

**Background:**

Burr hole craniostomy is a widely used method for the evacuation of CSDH. However it is not clear whether the irrigation during operation improves the prognosis or gives rise to additional complications instead. This retrospective cohort study was conducted to determine this issue.

**Methods:**

Patients attending two medical centers in China who underwent burr hole drainage with irrigation (BHDI) or burr hole drainage without irrigation (BHD) for unilateral CSDH during January 2013 to December 2016 were included in this study. The patients’ clinical information and follow-up data were retrospectively reviewed, and the radiologic findings were processed using the 3D Slicer software. The differences in outcomes were identified using *t-*test, chi-square test, or Fisher’s exact test.

**Results:**

A total of 151 patients comprising 63 patients in the BHD group and 88 patients in the BHDI group were included. Patients in the BHDI group had a higher volume of pneumocrania on the first postoperative day than that of patients in the BHD group (*p* < 0.05). No significant differences were observed between the two approaches in rates of rebleeding, recurrence and other complications (*p* > 0.05).

**Conclusions:**

Irrigation had no improvement in the long-term curative effect on CSDH, but it increased the risk of short-term complication in terms of pneumocrania. Therefore, this study suggests that irrigation is not an obligatory procedure during burr hole drainage.

**Electronic supplementary material:**

The online version of this article (10.1186/s12893-017-0295-x) contains supplementary material, which is available to authorized users.

## Background

Chronic subdural hematoma (CSDH) is a common clinical entity in neurosurgery, especially among elderly patients, with an incidence of about 5 per 1000 individuals per year [1]. The pathogenesis is inconclusive, and the optional treatments vary [2]. Although surgical therapy is generally accepted (e.g., twist drill craniotomy, burr hole craniotomy, and craniectomy), optimal surgical procedures remain controversial [3–7].

Burr hole craniostomy is a commonly chosen procedure for the initial treatment of CSDH [6, 7]; however, there are some differences among the operation details, for instance, two holes versus one hole, irrigation versus without irrigation, drainage versus no drainage, and irrigation with normal saline versus irrigation with drugs. All these procedures are used in contemporary clinical practice because there is no definite evidence indicating the most optimal treatment. Some studies compared the results between burr hole drainage without irrigation (BHD) and burr hole drainage with irrigation (BHDI) for the treatment of CSDH; however, the conclusions were controversial [7–11]. Therefore, we conducted this retrospective study to precisely determine this issue and compare these two surgical procedures in terms of complications and recurrence.

## Methods

### Patients

A total of 151 patients with unilateral CSDH who were admitted to the West China Hospital, Chengdu, and Union Hospital, Wuhan, in China and underwent surgery (BHD or BHDI) from January 2013 to December 2016 were included in this study. The datasets used in this study were de-identified. Eligible patients had to meet the following inclusion criteria: (1) a definite unilateral CSDH, (2) receiving one hole BHD or BHDI, and (3) availability of clinical data and follow-up information. The exclusion criteria were as follows: (1) patients with bilateral CSDH or subdural hydroma, (2) patients undergoing other surgical procedures and not one hole BHD or BHDI, and (3) patients whose clinical data and follow-up information were unavailable. We excluded patients receiving not one hole craniostomy because it might affect the comparability of some results, especially the volume of pneumocrania. A total of 156 patients met the inclusion criteria, but five of them were lost to follow-up after discharge, resulting in a final sample of 151 patients for this study.

BHD and BHDI were the most commonly used methods of operation to treat CSDH. In accordance with the preferences of surgeons, patients were classified into two groups according to a randomly allocated operative procedure: the BHD group (*n* = 63); and the BHDI group (*n* = 88). The clinical data and brain computed tomography (CT) scans for each patient were retrospectively reviewed, and the following variables were recorded: age, sex, affected side, concomitant diseases, preoperative hematoma volume, volume of pneumocrania (on the first postoperative day), rebleeding, residual hematoma volume (on the eighth postoperative day), infection (wound or intracranial), epilepsy, and recurrence (at 1-year follow-up period). The volumes of pneumocrania and hematoma were accurately calculated using the image-processing software (3D Slicer; Surgical Planning Laboratory, Brigham and Women’s Hospital, Harvard Medical School, Boston, MA, USA).

### Surgical technique and management

CSDH is a type of disease listed under the Clinical Pathway Program in China, and its management is standardized. All participants received standard of care treatment. Because there is no certain conclusion on the efficacy of irrigation, whether to irrigate is dependent on the individual surgeon preference. Symptomatic patients with CSDH were diagnosed based on a CT scan showing a typical crescent-shaped hematoma that exerted a mass effect on the midline structure. The operation was routinely performed by the neurosurgical team when patients exhibited an exact operative indication on the second day after admission. The surgery was primarily performed under local anesthesia with 10 ml lidocaine (1%), whereas irritable and intolerable patients were administered general anesthesia. Taking one-third of the posterior part of the thickest layer of hematoma as the drill point, the operation area was placed on the highest plane of the head to avoid pneumocrania. The surgical procedures were performed as follows. First, burred hole was done using standard craniotome of hand drill and a hole with a diameter about 1.5 cm was generated. The dura mater and the outer hematoma membrane were then dissected. Second, a standard ventriculostomy catheter (No. 5) was inserted to subdural space after spontaneous drainage of hematoma immediately after opening the dura in BHD. In BHDI, however, further repeated irrigation with warm saline was performed until the fluid became almost clear. Finally, the wound was sutured and the operation was completed. Closed drainage systems were applied to all patients in two groups. Both group received the routine postoperative treatment. Rechecking using CT scan was arranged on the first and eighth days after surgery, while the drainage tube was removed on the third day, and the patient was discharged on the ninth day after operation.

### Definition of recurrence

Patients showing reappearance of neurological symptoms with increasing hematoma volume on the operated side within 1 year after surgery and undergoing a repeated operation were considered to have recurrence [8].

### Statistical analyzes

All statistical analyzes were performed using the Statistical Package for the Social Sciences, version 19.0 (SPSS, Chicago, IL, USA). The *t-*test was applied to compare the differences among the measured data. Categorical variables were compared using chi-square test or Fisher’s exact test. *p* < 0.05 was considered to be statistically significant. The statistical power was calculated using the G*Power 3.1.9.2 software.

## Results

The patients’ demographic characteristics are summarized in Table 1. A total of 151 patients were included in this study, 88 in the BHDI group and 63 in the BHD group. There were 95 males and 56 females with an age range of 47–89 years (mean: 65.1 ± 7.0 years).

**Table 1:** Characteristics of patients who underwent burr hole drainage for chronic subdural hematoma

	BHDI group(*n* = 88)	BHD group(*n* = 63)	Total (*n* = 151)	Test value	P
Age(years)	65.5 ± 7.8	64.5 ± 5.6	65.1 ± 7.0	*t* = 0.843	0.401
Gender				χ^2^ = 0.652	0.419
Male (%)	53(60.2)	42(66.7)	95(62.9)		
Female (%)	35(39.8)	21(33.3)	56(37.1)		
Affected side				χ^2^ = 1.125	0.289
Left (%)	44(50.0)	37(58.7)	81(53.6)		
Right (%)	44(50.0)	13(41.3)	70(46.4)		
Concomitant diseases					
Hypertension (%)	18(20.5)	13(20.6)	31(20.5)	χ^2^ = 0.001	0.978
Organic heart disease (%)	13(14.8)	12(19.0)	25(16.6)	χ^2^ = 0.486	0.486
Diabetes (%)	11(12.5)	10(15.9)	21(13.9)	χ^2^ = 0.349	0.555
Preoperative hematoma volumes (ml)	51.42 ± 9.91	52.23 ± 10.21	51.76 ± 10.00	*t* = 0.494	0.622

*BHDI* burr hole drainage with irrigation, *BHD* burr hole drainage without irrigation

In the BHDI group (*n* = 88 patients), there were 53 males (60.2%) with a mean age of 65.5 ± 7.8 years. Half of the patients (44, 50.0%) were affected on the left side, and the volume of the preoperative hematoma ranged from 35.1 to 81.2 ml (mean: 51.42 ± 9.91 ml). Regarding comorbidities, 18 (20.5%), 13 (14.8%), and 11 (12.5%) patients had hypertension, organic heart disease, and diabetes, respectively.

The BHD group (*n* = 63 patients) had 42 males (66.7%) with a mean age of 64.5 ± 5.6 years. A total of 37 patients (58.7%) were affected on the left side, with a preoperative hematoma volume of 34.2–75.2 ml (mean: 52.23 ± 10.21 ml). Regarding the comorbidities, 13 (20.6%), 12 (19.0%), and 10 (15.9%) patients had hypertension, organic heart disease, and diabetes, respectively. No statistically significant differences were observed in the patients’ demographic data between the two groups.

**Fig. 1:**
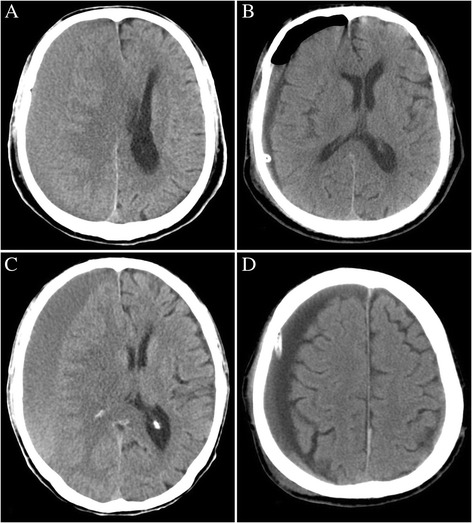
CT scans of two typical patients undergoing BHD and BHDI. **a** and **b** are the preoperative and first postoperative day images of a patient receiving BHDI, respectively. **c** and **d** are the preoperative and first postoperative day images of a patient receiving BHD, respectively. Obvious pneumocrania can be observed in image **b**. This image is from the preliminary report of the present study, which was published in West China Medicine Journal [27]. The authors have got the permission to republish this image

The duration of follow-up was at least 1 year. Five patients (three in the BHDI group and two in the BHD group) and two patients (one in each group) suffered from epilepsy and wound infection, respectively, with no statistically significant differences between the groups in these two factors. The volume of pneumocrania (on the first postoperative day) in the BHDI group (mean: 9.05 ± 3.50 ml) was significantly larger than that in the BHD group (mean: 4.85 ± 2.35 ml) (*p* = 0.001). The rate of rebleeding in the BHDI group (11/88, 12.5%) was higher than that in the BHD group (2/63, 3.2%), although the difference was not statistically significant (*p* = 0.085). There were also no statistically significant differences between the two groups in residual hematoma volume on the eighth postoperative day (10.76 ± 3.19 vs 11.68 ± 4.10 ml, *p* = 0.126) and recurrence rate (6.8% vs 7.9%, *p* = 0.794). The results are shown in Table 2, and examples of typical cases are shown in Fig. 1.

**Table 2:** Outcome of two procedures treating chronic subdural hematoma

	BHDI group	BHD group	Test value	P	Power
Pneumocrania *(ml)	9.05 ± 3.50	4.85 ± 2.35	*t* = 8.266	0.001	1.000
Residual hematoma*(ml)	10.76 ± 3.19	11.68 ± 4.10	*t* = 1.541	0.126	0.326
Rebleeding (%)	11(12.5)	2(3.2)	χ^2^ = 2.959	0.085	0.498
Recurrence (%)	6(6.8)	5(7.9)	χ^2^ = 0.068	0.794	0.041
Epilepsy (%)	3(3.4)	2(3.2)	χ^2^ = 0.006	0.937	0.021
Infection (%)	1(1.1)	1(1.6)	χ^2^ = 0.057	0.812	0.007

*BHDI* burr hole drainage with irrigation, *BHD* burr hole drainage; Pneumocrania *, Pneumocrania in 1st postoperative day; Residual hematoma*, hematoma volumes of the 8th postoperative day

## Discussion

CSDHs are one of the most common neurosurgical diseases, which primarily affect the elderly patients aged ≥65 years [12–14]. The incidence of CSDH is expected to increase owing to the global increase in the elderly population [15]. The pathophysiology of CSDH is still not clear. It usually follows a minor trauma to the brain due to a head injury, which results in bleeding of the parasagittal bridging veins that leads to a CSDH located between the brain and the dura. The CSDH then becomes covered by a thick outer membrane and a thin inner membrane. The outer membrane contains endothelial gap junctions and macrocapillaries with increased permeability that permit the leakage of blood into the cavity and enlargement of the hematoma [16, 17]. The treatment methods of CSDHs are based on the presence of symptoms and clinical or imaging signs of cerebral compression. Patients with small size or mildly symptomatic CSDHs are generally considered for conservative treatments, while those with large size or dominant symptomatic CSHDs require surgery [18, 19]. There are various surgical techniques to treat CSDHs, such as twist drill craniotomy, burr hole craniotomy, craniectomy, and embolization of the middle meningeal artery [20]. Several studies have reported about different operations for the treatment of CSDHs, but no final conclusion has yet been reached regarding the most effective and the safest scheme [4, 21, 22]. This situation reflects the dilemma of choosing the optimal procedure.

Burr hole craniostomy is the most commonly used technique for the treatment of CSDH [23]. However, there are several differences between the operation details, such as one hole or two holes, irrigation or no irrigation, and irrigation with normal saline or with artificial cerebrospinal fluid or thrombin, which are up to the preference of the attending neurosurgeon [24].

Some studies compared the efficacy and complications of BHD and BHDI in the treatment of CSDH. Suzuki et al. reported recurrence rates of 3.4% in the BHD group and 3% in the BHDI group [8]. In Kuroki’s series, the recurrence rates were 3.6% in the BHD group and 13.3% in the BHDI group. However, the abovementioned studies did not report significant differences in the recurrence rates [9]. Aoki found that intraoperative irrigation significantly reduced the recurrence rate (from 29.2 to 6.7%), but the use of irrigation had no impact on morbidity or mortality [10]. Jiang showed that double-hole BHDI resulted in the fewest recurrences compared with single-or double-hole BHD [11]. Thus, no conclusion was reached whether irrigation was necessary.

We conducted this retrospective study to compare these two types of surgical procedures. To our knowledge, the sample size of the present study was the largest among all the related studies, and we applied 3D Slicer software for assessing CSDH for the first time. We found that the volume of pneumocrania (on the first postoperative day) in the BHDI group (mean: 9.05 ± 3.50 ml) was significantly larger than that in the BHD group (mean: 4.85 ± 2.35 ml) (*p* = 0.001). The pneumocrania was the only factor that showed a significant difference in this study. During the process of irrigation, the air easily entered into the cavity, resulting in severe pneumocrania in almost every patient in the BHDI group. Although this issue could be alleviated through scientific position setting and a reasonable operation, the irrigation resulted in a significant higher volume of pneumocrania. No statistically significant differences were observed between the groups regarding recurrence, volume of residual hematoma, epilepsy, and wound infection. The rate of rebleeding in the BHDI group (11/88, 12.5%) was higher than that in the BHD group (2/63, 3.2%), though the difference was not significant (*p* = 0.085). We believe that the irrigation procedure, especially an inappropriate practice, might increase the risk of bridge vein bleeding. Thus we think irrigation might have increased the risk of rebleeding.

The present study did not observe significant differences in the recurrence rates between BHD and BHDI, which indicated that the two procedures had equivalent efficacy in long-term outcomes. Meanwhile, BHDI significantly increased the volume of pneumocrania and might increase the risk of postoperative rebleeding, which might result in a series of factors unfavorable to patients. First, the large volume of pneumocrania, especially tension pneumocrania, caused by irrigation, might result in some symptoms such as headache, irritability, and mental manifestations. It might also prevent the restoration of brain tissue and increase the probability of intracranial infections. Second, irrigation (especially rapid injection) might increase the bleeding risk of bridge veins and the primary bleeding point, which in turn increases the probability of recurrence. Finally, repeated irrigation might contaminate the cavity and the wound, which increases the risk of postoperative intracranial and wound infections. If patients suffer from intracranial infections, the treatment cycle, cost, and prognosis would not be optimistic. To summarize, irrigation had no improvement in the long-term curative effect on CSDH, but it increased the risk of short-term complications. Therefore, the present study provides moderate evidence that there is no need for irrigation during BHD operation.

3D Slicer is an intelligent image-processing software and has been widely used in the medical field in recent years [25]. It can precisely measure the volume of target area based on CT or magnetic resonance imaging data [26]. This study introduced 3D Slicer in the field of CSDH research for the first time. With the development of precision medicine, it will play an even more important part in the process of diagnosis and treatment of diseases.

This study has several limitations. First, the retrospective cohort observational design was prone to selection, performance, attrition, and detection bias. Second, the differences in the general condition of the included patients might cause confounding factors. Thirdly, the operation details differed between patients even in the same group due to the preference of surgeons, which might affect the results. Finally, the statistical powers were not strong enough regarding a number of factors due to insufficient sample size. Hence, well-conducted randomized clinical trials are warranted.

## Conclusions

Irrigation during burr hole craniostomy could effectively and rapidly evacuate the hematoma. However, the procedure had no improvement in the long-term curative effect on CSDH, and showed a higher probability to develop pneumocrania. Therefore, irrigation might not be a necessary procedure to treat CSDH.

## Additional file

Additional file 1: Raw data. (XLSX 19 kb)

## Abbreviations

BHD: Burr hole drainage without irrigation (BHD)
BHDI: Burr hole drainage with irrigation
CSDH: Chronic subdural hematoma
